# Evaluation of a mobile safety center’s impact on pediatric home safety behaviors

**DOI:** 10.1186/s12889-021-11073-4

**Published:** 2021-06-08

**Authors:** Leah Furman, Stephen Strotmeyer, Christine Vitale, Barbara A. Gaines

**Affiliations:** 1 University of Pittsburgh School of Medicine, Pittsburgh, PA USA; 2 Department of Pediatric General and Thoracic Surgery, UPMC Children’s Hospital of Pittsburgh, Pittsburgh, PA USA

**Keywords:** Mobile, Injury, Prevention, Pediatric, Safety

## Abstract

**Background:**

A Mobile Safety Center (MSC) provides safety resources to families to prevent pediatric injury. The primary objective of this study was to assess the impact of an MSC on home safety behaviors.

**Methods:**

We conducted a prospective observational study with 50 parents and guardians recruited at community events attended by an MSC. Participants completed a pre-test assessing demographics and home safety behaviors prior to participating in the MSC’s home safety educational program. We conducted follow-up with participants 4 weeks (follow-up 1) and 6 months (follow-up 2) after their visit to the MSC to reassess home safety behaviors. We used descriptive statistics in addition to Friedman, Wilcoxon sum-rank, and Fisher’s exact testing to analyze respondent demographics and changes in home safety practices. Friedman and Wilcoxon sum-rank testing was performed only for participants who completed all surveys.

**Results:**

Of our 50 participants, 29 (58%) completed follow-up 1, 30 (60%) completed follow-up 2, and 26 (52%) completed both. Participants were more likely to have a fire-escape plan at follow-up 1 than on the pre-test (*p* = 0.014). They were also more likely to have the Poison Control Hotline number accessible in their cellphone or near a home phone at follow-up 1 compared to the pre-test (*p* = 0.002) and follow-up 2 compared to the pre-test (*p* < 0.001). Families with at least one household member who smoked or used e-cigarettes at any point during the study (*n* = 16 for the total population, *n* = 9 for those who completed both surveys) were less likely to have more than two smoke detectors installed at home during the pre-test (*p* = 0.049). However, this significantly changed across timepoints (*p* = 0.018), and while 44.4% reported more than two detectors during the pre-test, 88.9% reported this at both follow-ups.

**Conclusions:**

Home safety education through an MSC positively changed some reported safety behaviors and maintained these changes at long-term follow-up. By encouraging the adoption of better home safety practices, education at an MSC may decrease pediatric injury rates.

**Supplementary Information:**

The online version contains supplementary material available at 10.1186/s12889-021-11073-4.

## Background

For children beyond infancy in the United States, unintentional injury is the leading cause of death and non-fatal injury [1]. Child injuries that occur at home may be preventable through the use of evidence-based home safety devices and practices. In a 2015 review article, Gielen et al. suggested several practical home safety behaviors that had been shown to be teachable to families [2], including: working smoke alarms with educational programs, fire escape planning, and safe storage of medications and poisons with childproof locks. Some of these practices have been associated with decreased injury rates, demonstrating that they are teachable and likely effective [2–4].

Although evidence supports behavior modifications for the prevention of childhood injuries, the most effective and accessible modality to disseminate this information has not been determined. Some hospitals created safety centers for the purpose of providing safety education. However, these centers may be unknown to families who have not experienced a child injury warranting a medical visit, or inaccessible for some families due to travel and financial concerns. Another strategy utilized pediatric waiting rooms to provide injury prevention education, finding that 93.5% of families made some positive changes at home afterwards [5]. However, this method again depends on engagement with a medical provider to access injury prevention education.

**Fig. 1:**
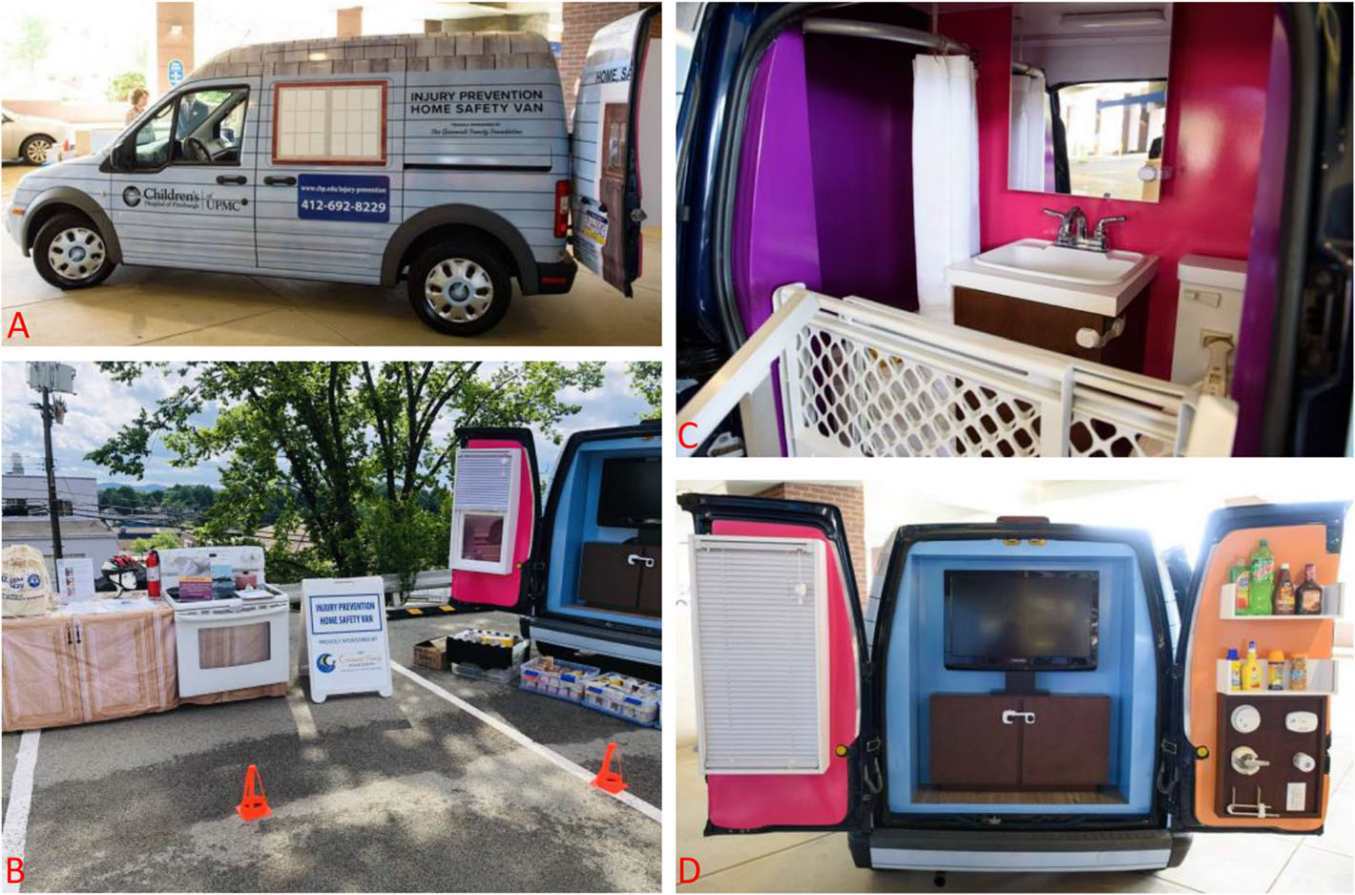
Pictures of the UPMC Children’s Hospital of Pittsburgh’s Mobile Safety Center. (**A**) Driver’s side exterior. (**B**) Setup at a community event, including packable kitchen model. (**C**) Passenger’s side interior, with bathroom model. (**D**) Rear interior, with living room model and safety item display

In contrast, a mobile safety center (MSC) can disburse home safety education and safety products in a diverse range of settings, including community events. MSCs are generally smaller-scale versions of safety centers designed to fit within a bus or van (Fig. 1). Two prior MSC studies suggested their utility; however, one study did not conduct participant follow-up [6], and the other did not mobilize the MSC, instead leaving it parked in a single location [7]. We previously published a study that examined the impact of an MSC on pediatric home safety knowledge and device use, recruiting participants through varied community events and settings and conducting follow-up with participants through 6 months post-visit. We found that visiting the MSC may increase home safety knowledge and the use of some freely distributed home safety devices, even at long-term follow-up [8]. We also found that by recruiting our study participants at community events, we successfully reached a lower socioeconomic status (SES) population. As low-SES is associated with higher rates of pediatric injury [9–17], it was important that we were able to provide education and resources for low-SES families.

During our prior study, we concurrently collected data regarding families’ baseline safety behaviors and the impact of the MSC on these behaviors. In this paper, we analyze and examine these results. Our behaviors of interest centered around fire, poison, and sharps safety, all of which were areas of safety intervention addressed by the MSC. We also examined the impact of a safety risk factor, smoking or e-cigarette use in the household [18–21], on fire safety behaviors, and the role that a child’s age may play in safety behaviors often targeted towards younger age groups. We hypothesized that at least some safety behaviors would change post-visit, that household smoking would not impact fire safety behaviors, and that households with younger children would be more likely to implement age-targeted behaviors.

## Methods

This was a prospective observational study approved by the University of Pittsburgh Institutional Review Board. Parents and guardians age 18 and older with children less than 18 years old living at home were recruited at six community events during June and July of 2018. All events were open to the general public, and participants were recruited by a study team member distributing flyers to all attendees. Written informed consent was obtained prior to engagement in research activities. Participants were surveyed at four time points: a pre-survey prior to MSC education including demographical questions, an immediate post-survey assessing safety knowledge only, and follow-ups at 4 weeks (“follow-up 1”) and 6 months (“follow-up 2”) post-visit. For a complete description of how participants were surveyed and contacted for subsequent follow-up, please see our prior publication on the impact of the MSC on home safety knowledge and device use [8].

Education at the MSC was conducted by trained injury prevention educators and centered around three home models: kitchen, bathroom, and living room (Fig. 1). At each event, the MSC was parked in an area that allowed for full, safe setup of all models, such as a parking lot or field. An educator was stationed at each model and demonstrated safety hazards in addition to correct safety behaviors and devices. As noted in our prior publication, this education was conducted either one-on-one with families or in small groups, lasted approximately 20 min, and covered safety in various areas of the home in addition to fire, firearm, fall, and poison/sharps safety [8]. All families received the same standardized core curriculum developed by the Safety Center at the Children’s Hospital of Pittsburgh (outlined in Supplement 1). While families could ask further questions or spend additional time with the models, this core material covered the knowledge and behaviors that we gathered data on during the course of our study.

**Fig. 2:**
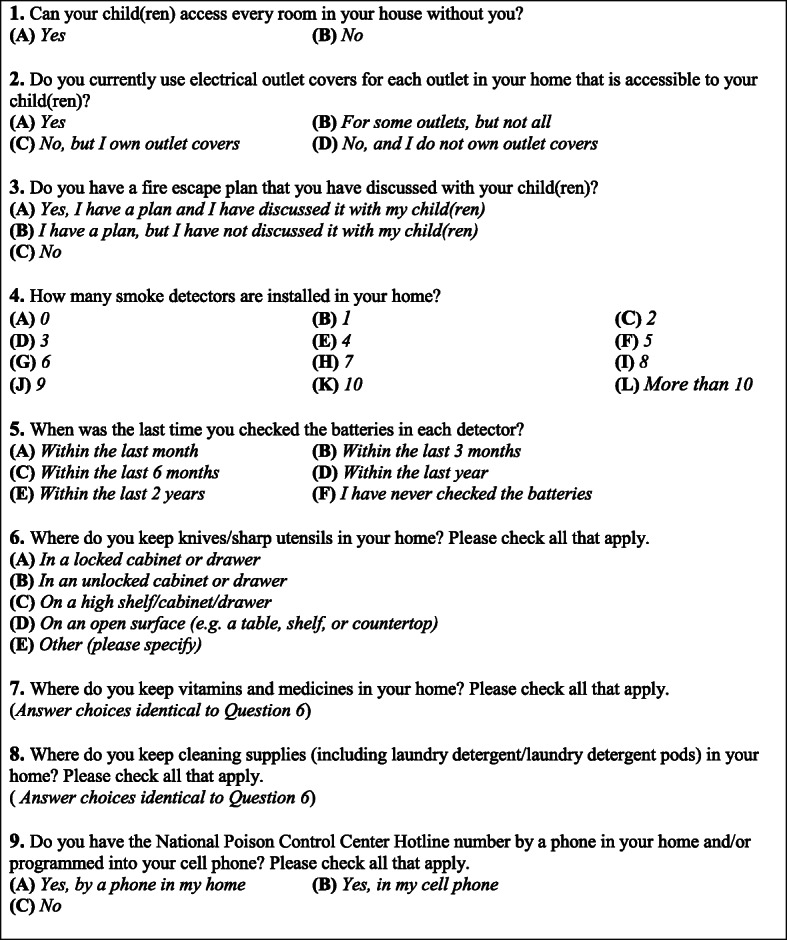
Questions and answer choices used to assess participant safety behaviors. Questions are numbered, answer choices are lettered and italicized

Questions and answer choices used to assess participant behaviors are presented in Fig. 2. These questions elicited whether a participant was using the “best practice” safety behavior: child(ren) unable to access every room, using electrical outlet covers for every outlet, having a fire escape plan and discussing it with child(ren), 3 or more smoke detectors per house (assuming that the parent(s) and child(ren) sleep in separate rooms, with one detector per bedroom and one outside), checking smoke detector batteries every month, storing poisons/sharps in a locked area, and having the Poison Control Hotline easily accessible in a cellphone and/or by the home phone. We also recognized that some of our participants may not use “best practice” behaviors but may still take “some precautions”: using electrical outlet covers for some outlets, having a fire escape plan but not discussing it with child(ren), 2 or more smoke detectors per house, and checking smoke detector batteries every 3 months.

We analyzed demographic and behavior data using frequency percentiles. We also used binary logistic regression analysis to determine if demographic factors were predictors of follow-up completion. To examine changes in behavior, we first selected all participants who completed both follow-ups (“complete follow-up group”). Next, we used Friedman testing to determine if those reporting a specific “best practice” behavior changed significantly between timepoints (pre-test, follow-up 1, and follow-up 2) We also conducted this analysis while examining participants reporting either a “best practice” behavior or “some precautions.” If the Friedman testing for a certain behavior indicated that there was a significant change, we then performed *post-hoc* Wilcoxon sum-rank (WSR) testing comparing timepoints in pairs to identify when significant change occurred (between the pre-test and follow-up 1, between follow-ups, and/or between the pre-test and follow-up 2). Friedman testing, followed by WSR testing if significant, was also used to examine changes in behavior among participants with at least one household member who smoked or used e-cigarettes in the complete follow-up group, and to examine changes in behavior among participants with at least one child 5 years of age or younger. Fisher exact testing was used to identify significant associations between whether at least one household member smoked and pre-test safety behaviors, and between whether a household included at least one child 5 years of age or younger and pre-test safety behaviors.

For all statistical analysis except *post-hoc* WSR testing, a *p*-value less than 0.05 was considered significant, and non-parametric tests were chosen given our small sample size. For *post-hoc* WSR testing, we applied the Bonferroni correction to our alpha level to decrease our rate of Type I error and considered a p-value less than 0.017 to be significant. In addition to *p*-values, Friedman testing and binomial logistic regression results are reported with chi-square (χ^2^), degrees of freedom (df, as χ^2^(df)), and odds ratio (binomial logistic regression only), and WSR testing results are reported with Z-scores (Z). Results were analyzed using IBM® SPSS® Statistics Version 25 (Mission Hills, CA).

## Results

### Demographics and home accessibility

While the number of participants at each event was not formally tracked, approximately 1/3 to 1/4 of event participants were eligible to and consented to participate in our survey, with an estimated total of 150–200 families who received education through the MSC across 6 events. Some examples of families who did not qualify for our study but did receive education included grandparents who provide intermittent childcare but did not have children living at their home and families expecting their first child.

Of our 50 surveyed participants, 29 (58%) completed follow-up 1, 30 (60%) completed follow-up 2, and 26 (52%) completed both. Some participant demographics, including gender, age, race/ethnicity, level of education, marital status, employment, and income level were previously reported in our prior study; notably, while female-predominant (90%), participant racial/ethnic breakdown resembled the population of the Pittsburgh area, while median annual income ($25,000 - $34,999) was lower than the local average [8]. The majority of participants were employed (56%), married or in a domestic partnership (60%), and 80% finished high school or held an equivalent degree [8]. Of the above-noted demographics, binary logistic regression revealed that only marital status was an independent predictor of follow-up completion (χ^2^(4) = 11.443, *p* = 0.022), with participants who were married or in a domestic partnership 5.23 times more likely to complete both follow-ups compared to single participants.

We also collected information about health insurance status, number of children in the home, number of adults in the home, and whether there was child injury in the home in the past year. Nearly all participants reported having adequate health insurance (96%). Most families (66%) had more than one child in the home, while only a minority of families (26%) had one adult in the home, and 20% reported a child injury at home in the past year. Binary logistic regression analysis showed that none of these factors predicted completion of both follow-ups.

With regard to home accessibility, 76% of all participants indicated on the pre-test that their child(ren) could access all rooms of their home, whereas 73.1% of the complete follow-up group did so. Friedman testing demonstrated that this did not change significantly across timepoints (χ^2^(2) = 0.500, *p* = 0.779).

### Fire safety

Fire, poison, and sharps safety behavior results are presented as a table comparing the results of all participants who took the pre-test (*n* = 50) to the complete follow-up group (*n* = 26) at three time points: the pre-test, follow-up 1, and follow-up 2 (Table 1). For most fire safety behaviors, the complete follow-up group was representative of the larger group of participants when comparing pre-test baseline behaviors within a 10% difference (Table 1). The only notable exception was “Covered every electrical outlet,” where 34.6% of the complete follow-up group reported this behavior compared to 46% of all participants (Table 1).

**Table 1:** Survey results for reported safety behaviors of participants and Friedman testing results comparing reported behaviors across timepoints in the complete follow-up group. All participants, *n* = 50. Complete follow-up group, *n* = 26. Friedman testing results reported with chi-squared values (χ^2^), degrees of freedom (df), and *p*-values. Significant p-values have been italicized and bolded; *p*-value < 0.05 was used to determine significance

Behavior	All participants, pre-test	Complete follow-up group, pre-test	Complete follow-up group, follow-up 1	Complete follow-up group, follow-up 2	Friedman results (χ^**2**^(df), ***p***-value)
***Fire safety***					
Covered every electrical outlet	46%	34.6%	57.7%	57.7%	χ^2^(2) = 5.556, *p* = 0.062
Covered at least some electrical outlets	64%	65.4%	80.8%	88.5%	χ^2^(2) = 4.222, *p* = 0.121
Made a fire escape plan	64%	61.5%	88.5%	80.8%	χ^2^(2) = 7.000, ***p = 0.030***
Made a fire escape plan, and shared this with child(ren)	48%	53.8%	57.7%	73.1%	χ^2^(2) = 4.000, *p* = 0.135
Installed 2 or more smoke detectors	86%	88.5%	92.3%	96.2%	χ^2^(2) = 2.000, *p* = 0.368
Installed 3 or more smoke detectors	70%	73.1%	84.6%	92.3%	χ^2^(2) = 5.429, *p* = 0.066
Checked the smoke detector battery within the last month	32%	23.1%	26.9%	38.5%	χ^2^(2) = 3.250, *p* = 0.197
Checked the smoke detector battery within the last 3 months	56%	53.8%	50%	69.2%	χ^2^(2) = 4.200, *p* = 0.122
***Poison and sharps safety***					
Stored sharps in a locked cabinet or drawer	12%	3.8%	7.7%	11.5%	χ^2^(2) = 1.500, *p* = 0.472
Stored medicines in a locked cabinet or drawer	18%	11.5%	7.7%	15.4%	χ^2^(2) = 2.000, *p* = 0.368
Stored cleaning supplies in a locked cabinet or drawer	40%	38.5%	23.1%	42.3%	χ^2^(2) = 4.667, *p* = 0.097
Made National Poison Control Center hotline readily accessible	38%	38.5%	76.9%	96.2%	χ^2^(2) = 23.333, ***p < 0.001***

Friedman testing showed that only one behavior demonstrated a significant change across timepoints: “Made a fire escape plan” (Table 1). Upon examining each pair of timepoints with *post-hoc* WSR testing, we found significant positive change between the pre-test and follow-up 1 (Z = 2.449, *p* = 0.014) but no significant changes between the pre-test and follow-up 2 (Z = 1.633, *p* = 0.102) or between follow-ups (Z = 1.000, *p* = 0.317).

There were a handful of behaviors that approached significant change across timepoints; namely, “Covered every electrical outlet” and “Installed more than two smoke detectors”. Both of these behaviors showed a positive trend when comparing the pre-test to both-follow-ups (Table 1). However, further examination with WSR testing was not completed as they failed to meet Friedman testing significance criteria.

### Subgroup analysis: household smoking

We explored whether household smoking impacted reported fire safety behaviors. When comparing baseline pre-test data from participants who reported at any surveyed timepoint that at least one household member smoked or used e-cigarettes (*n* = 16) to all other participants with Fisher’s exact testing, we found that households with at least one member who smoked were less likely to have more than two smoke detectors installed (50% vs. 79.4%, *p* = 0.049). However, when examining this subgroup within the complete follow-up group (*n* = 9), we found that these households significantly changed this behavior per Friedman testing (χ^2^(2) = 8.000, *p* = 0.018). While they were more likely to have more than two smoke detectors installed at both follow-ups (44.4% during the pre-test vs. 88.9% at both follow-ups), *post-hoc* WSR testing failed to meet significance (Z = 2.000 and *p* = 0.046 for both follow-ups). This subgroup also demonstrated a change in outlet safety, with Friedman testing revealing significant change when examining the use of outlet covers on all accessible outlets (χ^2^(2) = 7.600, *p* = 0.022). However, while participants increased use between the pre-test and follow-up 2, (22.2% vs. 44.4%), *post-hoc* WSR analysis was not significant (Z = 2.236, *p* = 0.025).

### Poison and sharps safety

The complete follow-up group did not differ more than 10% from all participants on the pre-test for any of the poison and sharps safety behaviors (Table 1).

As with the fire safety group, Friedman testing revealed that only one behavior significantly changed across timepoints: “Made National Poison Control Center hotline readily available,” which we defined as having written down by a phone in the household or programmed into a cell phone (Table 1). *Post-hoc* WSR testing demonstrated that follow-up 1 increased from the pre-test (Z = 3.162, *p* = 0.002), as did follow-up 2 (Z = 3.873, *p* < 0.001). Interestingly, follow-up 2 also increased in comparison to follow-up 1, although this did not meet *post-hoc* WSR significance criteria (Z = 2.236, *p* = 0.025).

### Subgroup analysis: families with young children

We wondered whether a child’s age impacted the accessibility of their home, the use of safety devices, and parental behaviors and beliefs, such as: electrical outlet covers, storage of sharps, medicines, and cleaning supplies, perceived safety, and parental concern for specific safety hazards. Through Fisher’s exact testing, we found that the baseline pre-test reports for these behaviors and concerns were not significantly different between households with at least one child 5 years of age or younger (*n* = 39) as compared to families with older children. Additionally, we examined this subgroup of families with at least one child 5 years of age or younger within our complete follow-up group (*n* = 19). For the above-mentioned behaviors and concerns, there were no significant changes across timepoints when examined via Friedman testing.

## Discussion

This study examined the impact of an MSC on reported home safety behaviors among participants recruited from community events. Reported adherence to ideal safety behaviors varied widely, though some behaviors positively changed following a visit to the MSC.

### Demographics and accessibility

Our previous work found that our study population was of lower-SES than the general population of Pittsburgh, with lower income, a lower percentage of high school graduates, and a lower attainment of higher-level degrees [8]. In this work, we validated our use of the complete follow-up group by finding that only one demographic, marital status, predicted completion of both follow-ups. We speculate that this may be due to the ability to rely on a spouse or domestic partner for childcare while the participant completed the study.

Most participants lived in houses which were completely accessible to their children. This may reflect a difficulty in completely restricting access to certain areas of the house, possibly through inadequate use of safety devices such as baby gates and doorknob covers. However, though families were offered free doorknob covers and cabinet latches at the MSC, the reported accessibility of the home to children did not significantly change post-visit, raising the concern that families may have had difficulty using or installing the provided safety devices to restrict access. Future work to assess barriers to implementation of devices and parental attitudes towards home accessibility may be beneficial to tailoring educational efforts.

### Fire safety

Participants were significantly more likely to have a fire escape plan after visiting the MSC; however, this change was only significant between the pre-test and follow-up 1. This indicates that there was a change that occurred after visiting the MSC, but this change may not have been fully sustained 6 months later. Interestingly, while participants were more likely to have a plan after their visit, they were not more likely to have discussed this plan with their children. This may have been due to factors such as parental belief that their child does not need to know the escape plan, or that it is unlikely that there will be a fire.

There were two fire safety behaviors that approached significant change: usage of electrical outlet covers on all accessible outlets and having more than two smoke detectors installed. Notably, our studied population was very small, and it is possible that we did not reach significance for the behaviors that approached significance due to under-powering. Unfortunately, almost half of all participants had last checked detector batteries over 3 months ago, and this did not change significantly across timepoints. This is worrisome, as even if participants installed more detectors after the visit, this would not necessarily increase safety if the detectors did not work.

Of note, we found that participants who reported that at least one household member smoked or used e-cigarettes differed significantly from all other participants in that they were initially less likely to have more than two smoke detectors installed. This was concerning, as there is a demonstrated association between smoking and fire risk [18, 19] and reported cases of fires and explosions linked to malfunctioning batteries within e-cigarettes [20, 21]. However, within this subgroup of participants in the complete follow-up group, there was a significant change in the number of detectors across timepoints. These households were more likely to have more than two smoke detectors installed at both follow-ups, although this increase did not meet *post-hoc* significance criteria. Additionally, this subgroup significantly changed their electrical outlet safety. They reported more electrical outlet covers on all accessible outlets on the pre-test compared to follow-up 2, although as with the change in detectors, this increase was not statistically significant.

Though the subgroup was small and our results limited in power, as demonstrated by the significant change shown during initial Friedman testing without significant *post-hoc* WSR results, this was an unexpected result of the analysis, as we did not expect the risk factor to potentially impact adoption of new safety behaviors. It is not entirely clear why these changes only occurred in the households with at least one member who smoked. As with implementing a fire escape plan, parental beliefs may have been a significant influencing factor in these decisions, and future work to assess reasoning and attitudes may be helpful when designing new educational strategies, perhaps using qualitative methods and parental focus groups.

### Poison and sharps safety

Of all the data collected during this study, we found the storage of medicines, sharps, and cleaning supplies to be the most surprising and disappointing. Very few families used locks to store sharps and medicines, and while more reported usage of locks for cleaning supplies, this still accounted for less than half of our participants. Unfortunately, these numbers did not significantly change during follow-up, despite the free provision of cabinet latches by the MSC.

By contrast, there was a significant increase in the number of families who reported easy accessibility of the Poison Control Hotline. Nearly all (96.2%) of the complete follow-up group had this number either in their cell phones or near a home phone by follow-up 2. Interestingly, this demonstrated a stepwise increase from follow-up 1. Though this increase was not significant on *post-hoc* testing, this may indicate that participation in the study itself may have been a stimulus for this safety behavior, perhaps by reminding participants when asked during follow-up 1.

The ease of changing these two behaviors may also have impacted their relative adherence. It may be difficult for families to lock up dangerous household items or to install safety locks or latches, but comparatively simpler to program the hotline number into a cell phone, or to write it down near a home phone. Again, qualitative assessment of the barriers to change may be beneficial to the creation of new, highly effective educational strategies.

### Subgroup analysis: families with young children

We previously suspected that the age of children in the home impacted certain safety behaviors that are primarily geared towards protecting younger children; namely, home accessibility, electrical outlet covers, and locked sharps, medicines, and cleaning supplies. Interestingly, whether a family had a child 5 years of age or younger at home did not significantly impact these behaviors at baseline on the pre-test. Furthermore, families with a child 5 years of age or younger at home did not significantly change these behaviors after visiting the MSC. This suggests that families may continue some safety behaviors even with older children, such as the 64% of all families who used electrical outlet covers on at least some outlets. Alternatively, some families may not engage in a certain behavior at any timepoint, illustrated by the 88% of all participants who did not lock away their sharps. This may be due to the difficulty of stopping some behaviors as compared to the difficulty of starting other behaviors; for example, it may be easier to leave electrical outlet covers in the socket once they are already there, but more challenging to initially lock up dangerous household products.

### Future directions and applicability in the COVID-19 era

As noted above, we did not track the number of event attendees, although only about 1/3 to 1/4 of MSC visitors participated in our study. A portion of non-studied visitors did not meet study criteria (e.g., grandparents who provided childcare or expectant parents) but benefitted from pediatric home safety education, as they had children frequently in their homes or expected to do so in the near future. By partnering with local community programs and centers, we were able to successfully reach a lower-SES population. However, some of MSC events were better attended than others, and attendance appeared to depend on how well the event was advertised by community partners. To augment event participation, it may be necessary to provide community partners with standard advertising materials for the MSC, which may highlight the hands-on nature of MSC education, free provision of some safety items, and even key study results. The formation of relationships with new community partners could also augment the scope of the MSC through a broader schedule of events.

Additionally, although this paper was conceived of prior to restrictions driven by COVID-19 precautions, it is important to note that the MSC can be easily adapted to provide community safety education and resources with such precautions in mind. Families can be given one-on-one education while outside wearing masks and socially distanced. For example, the picture shown in Fig. 1B was taken at an event after the start of the COVID-19 pandemic, with the orange safety cones marking safe distances away from the tables staffed by MSC team members. While many families may be particularly reluctant to visit a hospital setting, where many safety centers are located, the MSC presents a simple, safe alternative. Highlighting the COVID-19 precaution-friendly setting in community flyers may also boost event participation.

### Limitations

There are several limitations of note to our study. First, as a survey-based study, social desirability bias and responder bias may have adversely affected the validity of responses. Additionally, we conducted convenience sampling subject to participation bias at community events, did not formally track the number of event participants, and recruited a small number of participants, 40% of whom did not complete one or both of the follow-ups. While it is reassuring that our complete follow-up group closely resembled our original sample, which in turn appeared to represent a lower-SES section of the local population, many of our non-significant results may have been under-powered. Finally, our study demonstrated significant positive changes for some safety behaviors, but we did not study the impact of these changes on injury rates, and more work is necessary to determine if these changes make significant clinical impacts.

## Conclusions

We found that visiting the MSC increases adherence to some home safety behaviors. Use of MSCs could reduce pediatric injury rates by encouraging such practices within the community. Adherence to certain fire safety behaviors was impacted by whether a participant had at least one household member who smoked. Safety behaviors primarily aimed towards younger children were not more likely to be observed among families with children 5 years and younger. Some safety behaviors, specifically locking dangerous household items, had low adherence which did not significantly change after visiting the MSC, indicating that the MSC’s safety curriculum may benefit from more education and emphasis on these behaviors in the future.

## Supplementary Information

**Additional file 1.**

## Data Availability

The datasets used and/or analyzed during the current study are available from the corresponding author on reasonable request.

## Abbreviations

MSC: Mobile Safety Center
SES: Socioeconomic status
WSR: Wilcoxon Sum-Rank
χ^2^: Chi-square
df: Degrees of freedom
Z: Z-score
