# Supplementary material for: Evaluation of a mobile safety center’s impact on pediatric home safety behaviors
Source: BMC Public Health. 2021 Jun 8;21:1095. doi: 10.1186/s12889-021-11073-4 (PMC8184352; doi:10.1186/s12889-021-11073-4)
Supplement: Supplementary file 1 — Additional file 1. [file 12889_2021_11073_MOESM1_ESM.docx]

SAFETY CENTER CURRICULUM

- Infants < 1 year – Safe Sleep Education
  - 2 weeks – ¾ months - Purple Crying Education
- Children < 8 years – Car seat Information
- E-cigarette Use – Educate about safely storing vaping liquids
- Driveway – Educate about looking around to avoid rollovers
- Most of our products are for kids under 5 years
- REMEMBER: Things can be child resistant, but NOTHING is child ‘proof’
  - Want layers of protection

# Bedroom

- Baby sleeps alone, every nap, every night (includes no soft bedding or stuffed animals)
- Lay babies on their back
- Take note of what they can reach from their crib (lamp, cords)
- Smoke alarm – outside every bedroom, one on every floor, check once every month, replace every 10 years (expiration date)
- Carbon Monoxide – one on every floor, 15 feet away from ‘source’ (has expiration date)
- **Resources:** sleep sacks, smoke detectors

# Kitchen - Poison, Choking, Sharps, Burn Prevention

- Make sure cleaning supplies are locked and stored away
  - Every day, over 300 children in the United States ages 0 to 19 are treated in an emergency department, and two children die, as a result of being poisoned
  - Medications locked and stored away
    - Including visitors’ medications
    - Don’t call Medicine “Candy”
    - Be aware of ‘nontraditional’ poisons
      - Vitamins, gummy melatonin, make up, hair care supplies,
  - Poison control number near phones, app is available, number for pets too
  - If unsure make the call
    - 1-800-222-1222
- Children under 5 should not eat small, round, or hard foods including pieces of hot dogs, cheese sticks or chunks, hard candy, nuts, grapes, marshmallows, or popcorn
  - Be aware of small toys that are choking hazards as well
    - Jacks, marbles, monopoly pieces, etc
- Knives and sharp utensils should be locked and stored away
- Every day, 300 children ages 0-19 are treated in emergency departments for burn related injuries
  - Keep pots and pans on the back burner, handles turned in
  - Stove knob covers available, create a hot zone – 3 feet around stove so kids know not to step in
  - Create fire escape plan (2 ways out of every room, meeting place), place on fridge, practice so everyone knows where to go, kids know not to hide
  - Make sure Candles and Lighters are out of reach of little hands
- Button batteries extremely dangerous – keep away from small children
  - Found in cards that sing and dance, toys like fidget spinners, and remotes
  - If you think your child has swallowed one (look like candy), no food or water, immediately call 911, is a medical emergency, don’t induce vomiting
- Rare earth magnets – super strong, marketed to adults
  - If you think your child has swallowed one (look like candy), no food or water, is an emergency
- **Resources:** Choke tube, wind-ups, latches, knob covers, medication boxes, oven locks, stove knobs, fire extinguishers

# Bathroom

- NEVER leave a child unsupervised near water (tub, toilet, buckets, ponds, pools)
  - Children can drown in an inch of water
  - Drowning can occur in seconds
  - Drowning is the leading cause of death for children 1-4 years old.
  - If unable to locate your child always check the water first (pool, fountain, tub, toilet, etc.) Seconds count.
  - Your child is also more likely to drown in the backyard pool or hot tub.
- **Resources:** Spout cover, thermometer, toilet lock, doorknob cover
- Keep hot water tank temperature at 120 degrees or less
  - Takes more than a few seconds to get hot
- Unintentional falls are the leading cause of non-fatal injuries for children in the U.S.
  - Place bathmat outside of the tub helps the whole family
  - Spout cover to prevent head injuries
- **Resources:** tub thermometer (duck), spout cover
- Windows should not be opened more than 4 inches at the bottom (open from the top if possible or use window wedges)
  - Do not place furniture under windows
  - Screens are not designed to keep children in, only to keep insects out
  - Make sure window cords are up and out of the way for kids
  - About eight children die each year after becoming entangled in a window cord and 8 more die from window falls each year
- **Resources:** window cord wind up, window lock
- Electrical outlet covers over every outlet
- **Resources:** outlet covers

# Stairs & Falls

- Keep clutter away from stairs, use safety gates at the bottom and top of stairs
- The gates are pressure mounted, the top of stairs need to be screwed into the wall, we do not want the gate to fall down on top of kids
- Every 6 minutes, a child younger than 5 is rushed to an ER for a stair-related injury
- We have furniture anchors to prevent heavy furniture from toppling over
  - Bedrooms are high risk places for furniture to fall onto children, more than half of all TV tip overs occur in bedrooms
- Use tv straps for televisions to keep from toppling over
  - Recommend mounting them to the wall
- About every 43 minutes a child in the U.S. is injured from a TV or furniture tip-over incident.
- **Resources:** baby gate, furniture anchors

# Firearm Safety

- - Store guns in a locked location (gun safe, vault, etc.) unloaded, out of reach and sight of children
  - Store ammunition in a separate locked location, out of reach and sight of children
  - Keep the keys and/or combination hidden
  - When a gun is not in a lock box keep it in your sight at all times
  - Be sure all guns have a childproof lock
  - Looking at statistics, teenagers are impulsive and access to a gun increases the risk of death by suicide by 3 times
  - It is likely that your children know where your gun is kept, don’t assume it’s a secret
  - ASK if there are guns where your child plays (friends’ home, other family, etc.) and how they are stored – if you aren’t comfortable with the answer suggest an alternative plan for play
    - Children as young as 3 years old are strong enough to pull a trigger.
